# Response to the letter to the editor

**DOI:** 10.1093/ehjcr/ytaf187

**Published:** 2025-04-11

**Authors:** Felipe Israel López-Trejo, Elias Noel Andrade-Cuellar

**Affiliations:** Clinical Cardiology, National Medical Center ‘November 20th’, ISSSTE, Av. Felix Cuevas #540, Col. Del Valle Del. Benito Juarez, Ciudad de México, C.P. 03100, Mexico; Universidad Nacional Autónoma de México, Escolar 411A, Copilco Universidad, Coyoacán, 04360 Ciudad de México, Mexico; Clinical Cardiology, National Medical Center ‘November 20th’, ISSSTE, Av. Felix Cuevas #540, Col. Del Valle Del. Benito Juarez, Ciudad de México, C.P. 03100, Mexico; Cardiac Electrophysiology, National Medical Center ‘November 20th’, ISSSTE, Av. Felix Cuevas #540, Col. Del Valle Del. Benito Juarez, Ciudad de México, C.P. 03100, Mexico; Universidad Nacional Autónoma de México, Escolar 411A, Copilco Universidad, Coyoacán, 04360 Ciudad de México, Mexico

To the Editor,

Takotsubo syndrome (TTS) may present with atypical triggers such as electrolyte disturbances, posing diagnostic and therapeutic challenges. Our recently published case of hyponatremia-induced TTS secondary to idiopathic syndrome of inappropriate antidiuretic hormone secretion (SIADH) provides several key educational points.^1^

Monitoring serum sodium daily is essential, even in patients with normal initial values, as evolving hyponatremia may worsen both neurological and cardiovascular outcomes.^2^ Early detection and appropriate correction of sodium levels are crucial to prevent clinical deterioration in TTS. In cases of suspected SIADH, urine osmolality and sodium should be assessed prior to hypertonic saline administration and re-evaluated during correction to confirm diagnosis and guide therapy.^2^

Identifying underlying causes is fundamental. In our patient, an extensive workup (oncological, infectious, and endocrinological) revealed no etiologic condition, supporting the diagnosis of idiopathic SIADH.^3^ Although pregabalin has been implicated in drug-induced SIADH in two previous reports,^4,5^ our patient had been on stable pregabalin therapy for 5 years without prior sodium disturbances. A Naranjo algorithm score of 0,^6^ and the absence of recurrence or cardiac deterioration during follow-up despite ongoing treatment, made this association unlikely in our case.

Pregabalin’s mechanism as an alpha2-delta ligand that modulates calcium channels and reduces neurotransmitter release offers a plausible, though unproven, pathway linking it to TTS. This highlights the need for further research and reinforces the utility of tools like the Naranjo algorithm in differential diagnosis.^5^

Cardiac magnetic resonance (CMR) plays an important prognostic role in TTS. Late gadolinium enhancement (LGE) is reported in 22.4–40.7% of TTS patients, depending on timing,^7^ and reflects localized myocardial inflammation and focal oedema due to extracellular matrix expansion. In our case, CMR revealed myocardial oedema without ischaemic injury or transmural necrosis. We did not perform T1 mapping or extracellular volume fraction measurements, given that LGE was already present. However, these tools are especially useful when LGE is absent or gadolinium is contraindicated.^8^

LGE in TTS has been associated with prolonged ventricular recovery and delayed electrocardiographic normalisation,^9^ which may reflect persistent myocardial inflammation. Although speculative, hyponatremia could potentially contribute to this process, warranting further investigation.

In summary, hyponatremia should be addressed proactively in TTS, as it may act as both a primary trigger and a secondary exacerbating factor. The use of diagnostic algorithms and advanced imaging tools is key to refining differential diagnoses, understanding pathophysiological mechanisms, and optimising clinical outcomes (Figure 1).

**Figure 1:**
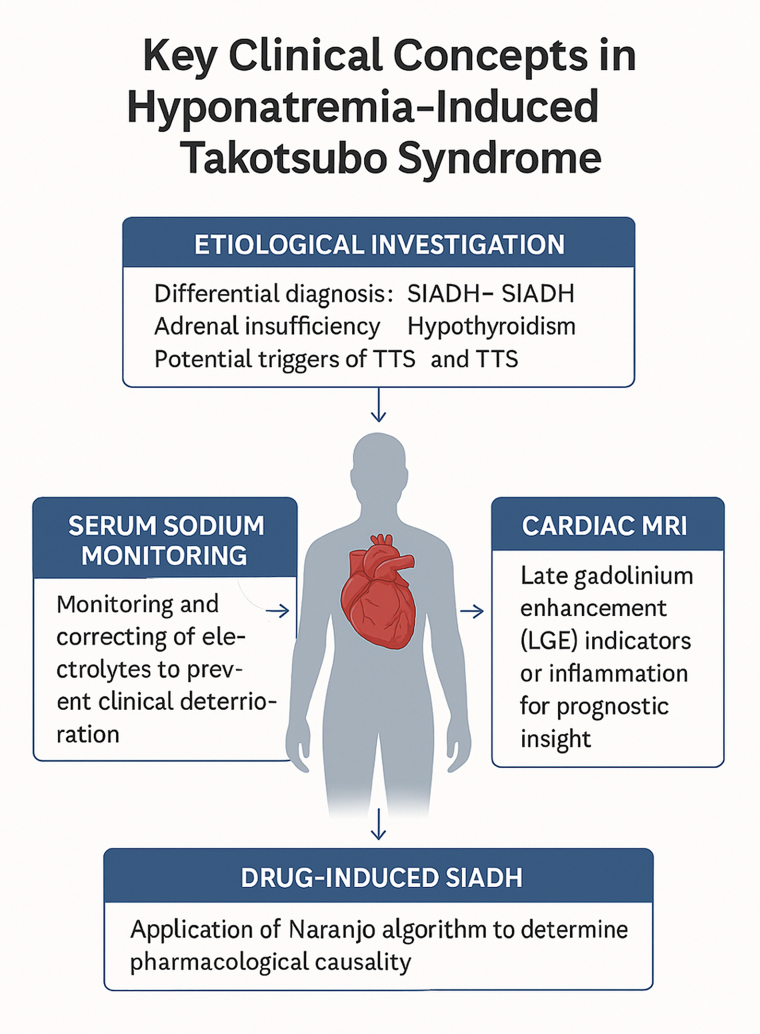
Key clinical concepts in hyponatremia-induced Takotsubo syndrome.

## Data Availability

The data underlying this article are available in the article and in its online Supplementary material.

## Notes

*Consent*. Informed consent was obtained from the patient in accordance with COPE guidelines.
